# Human cytomegalovirus infection and colorectal cancer risk: a meta-analysis

**DOI:** 10.18632/oncotarget.12523

**Published:** 2016-10-08

**Authors:** Bingjun Bai, Xingxing Wang, Engeng Chen, Hongbo Zhu

**Affiliations:** ^1^ Department of Colorectal Surgery, Sir Run Run Shaw Hospital, School of Medicine, Zhejiang University, Hangzhou, China; ^2^ Key Laboratory of Biotherapy of Zhejiang province, Hangzhou, China

**Keywords:** HCMV, colorectal cancer, viral DNA, tumor progression, meta-analysis

## Abstract

Human cytomegalovirus infection (HCMV) has been recently considered as a factor for tumorigenesis. The current study used meta-analytical techniques to explore the prevalence of HCMV in tumor tissues and the relationship between human cytomegalovirus and colorectal cancer (CRC) risk. 11 studies detecting HCMV DNA in tumor tissues were included in meta-analysis. The prevalence rate and odds ratio (OR) were two main parameters. The overall prevalence of human cytomegalovirus DNA in tumor tissues were 27.5% (95% CI = 17.2%−37.8%). Binary logistic regression showed that the studies reported before 2010 involving formalin-fixed specimens from patients in developed region represented a lower proportion of HCMV. The tumor tissues had a significantly higher rate of virus infection compared with normal tissues (OR = 6.59, 95% CI = 4.48−9.69, *I*^2^ = 0%, *P* = 0.71). Subgroup analysis revealed the prevalence of the virus didn't differ in patients with different tumor stages, in tumor cells with different histologic grades, also in different kinds of specimen (polyp and adenocarcinoma). The results of current study suggested a statistically association between the virus infection and an increased risk of colorectal cancer.

## INTRODUCTION

Human cytomegalovirus, also known as human herpesvirus-5 (HHV-5), is a ubiquitous opportunistic herpesvirus that infects between 60% and 70% of adults in developed countries and almost 100% in developing countries. As one of the pathogens, HCMV is able to induce the symptoms characterized by sore throat, prolonged fever and mild hepatitis [1]. Of course, a latent infection without any symptom is a more common situation. HCMV remains life-long infection and can be reactivated at any time, eventually causing significant morbidity and even death, especially in immunocompromised hosts [2]. Currently, increasing evidence has highlighted the relationship between HCMV infection and human cancers. HCMV genomes or gene products have been detected in several cancers including malignant glioma, cervical carcinoma, Kaposi's sarcoma and breast cancer [3–6]. *In vitro* studies have verified the capacity of HCMV for transforming cells and increasing tumourigenicity [7]. However, unlike other DNA viruses playing a role in human malignancies by specific viral genes, HCMV often presents no specific and even undetectable DNA sequence in transformed cells, which can be explained by “hit and run” mechanism [8, 9]. In recent years, the oncomodulatory potential of HCMV has been noted according to the data from studies with tumor cell lines infected by HCMV. Oncomodulation allows HCMV to increase malignancy of infected tumor cells by regulating signal pathways, transcription factors and tumor suppressor proteins [10, 11].

Colorectal cancer is globally one of the leading causes of cancer-related mortality. It shows a five-year survival rate less than 60% in Europe [12]. The pathogenesis of CRC has been long focused on environmental factors such as insufficient activity, high-fat diets, smoking and living in a developed country [13]. Recently increasing attention has been paid to the uncertain but significant effect the infection of HCMV as a microorganism exerts on CRC. Since HCMV nucleic acid was first detected in tumor tissues of CRC patients, the similar results had been confirmed by other studies [14]. Some controlled studies have supposed that the prevalence of HCMV DNA or proteins differs significantly between tumor tissues and matched normal tissues [15, 16]. The presence of human cytomegalovirus in tumor specimens is even related to prognosis of patients with CRC [17].

Nevertheless, detection of HCMV in CRC remains controversial. Negative results indicate that HCMV may present lack of association with CRC regardless of type of tissues or detection methods [18]. Inadequate data has been obtained to confirm the causal role of HCMV in carcinogenesis processes, tumor progression and metastasis [19]. Although HCMV infection is considered to have relationship with CRC, the clinical and pathological features of patients are also various [20, 21].

We pooled the original literature concerning detection of HCMV in CRC tissues and performed a meta-analysis to investigate the relationship between HCMV infection and CRC patients and their clinicopathological characteristics.

## RESULTS

**Figure 1:**
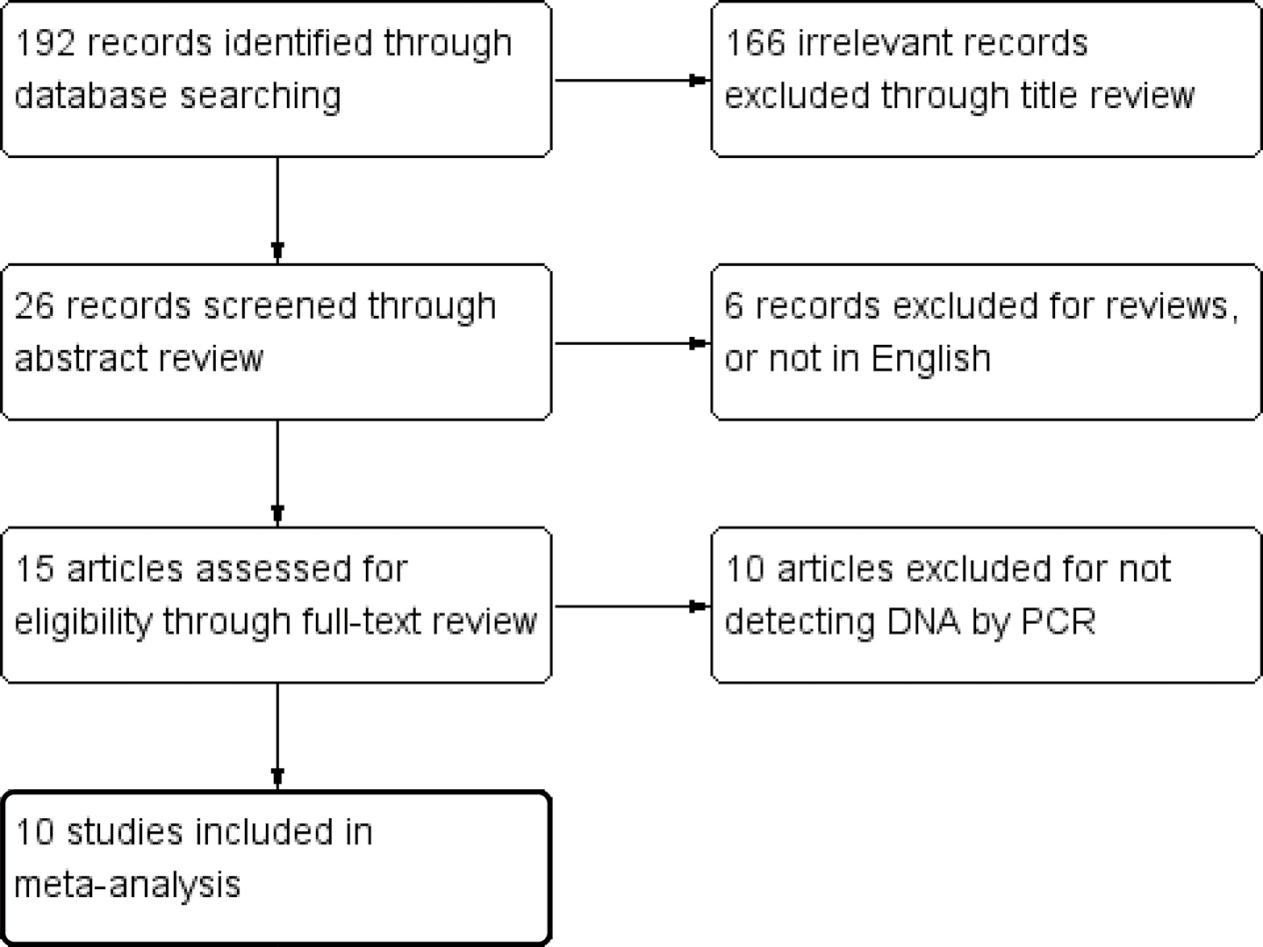
Flow diagram of search strategy

The literature search resulted in 192 relevant articles after removing the duplicate ones. By carefully screening the title of these articles, 166 were excluded for not focusing on the desired topic. The criteria of inclusion and exclusion further excluded 16 articles (4 reviews, 2 not in English and 10 without detecting DNA by PCR) thus 10 qualified articles were included (Figure 1) [15–17, 19–25]. One of these articles had separately studied two different regions (Sweden and Vietnam) of patients [16], so it was regarded as two individual articles and labelled as “Dimberg, J. 1” and “Dimberg, J. 2” respectively. Finally, the number of all trials involved was 11. The year of publication ranged from 2004 to 2016. Among these studies, 3 studies were performed in European patients and 6 in Asian patients. 1 study investigated patients in USA, while 1 in South America. For all 11 studies, 4 of them brought into analysis matched normal tissues from the same patient with CRC as a control group. Characteristics of all included studies were summarized in Table 1.

**Table 1:** Detailed information of included studies

Study No.	Publication year	First author	Regions	Type of samples	No. of cases	No. of controls
1	2013	Dimberg, J. 1	Sweden	fresh/frozen	119	119
2	2013	Dimberg, J. 2	Vietnam	fresh/frozen	83	83
3	2014	Tafvizi, F.	Iran	formalin-fixed	42	-
4	2015	Chen, H.P.	Taiwan	fresh/frozen	115	115
5	2012	Chen, H.P.	Taiwan	fresh/frozen	163	163
6	2016	Chen, H.P.	Taiwan	fresh/frozen	83	-
7	2013	Chen, H.P.	Taiwan	fresh/frozen	95	-
8	2009	Bender, C.	Italy	formalin-fixed	36	-
9	2008	Mariguela, V.C.	Brazil	fresh/frozen	14	-
10	2004	Knösel, T.	Germany	fresh/frozen	57	-
11	2005	Akintola-Ogunremi, O.	USA	formalin-fixed	13	-

### The prevalence of HCMV infected CRC

**Figure 2:**
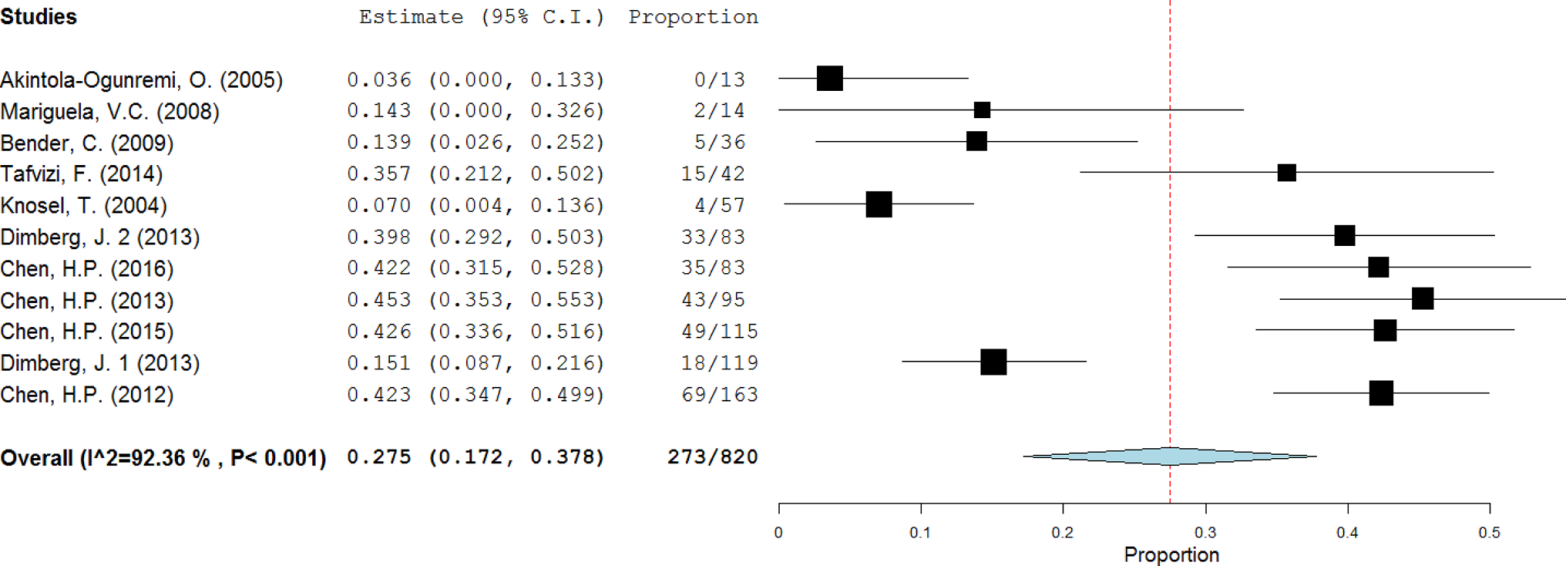
The prevalence of HCMV in CRC tissues A random-effects model was performed.

Meta-analysis revealed considerable heterogeneity in positive rate of HCMV infection in patients diagnosed with colorectal cancer with the overall prevalence 27.5% (95% CI = 17.2%–37.8%) (Figure 2). The HCMV infection in cases with different features of patients and specimens were present (Table 2). The lowest prevalence of HCMV-infected CRC was in patients of USA, about 3.6% (95% CI = −6.1%–13.3%). And it was much higher in Asia subgroup 42.0% (95% CI = 37.9%−46.0%). Considering Europe and USA as a developed region, Asia and Brazil as a developing region, the former demonstrated a significantly lower positive proportion than the latter (aOR = 0.257, 95% CI = 0.159–0.414). As for the year of publication, the studies reported before 2010 presented a low prevalence of HCMV 7.9% (95% CI = 3.1%−12.7%), which was significantly lower than the studies published after 2010 (aOR = 0.488, 95% CI = 0.237–1.005). Compared to the fresh and frozen specimen, formalin-fixed specimen presented a markedly lower prevalence of HCMV DNA (aOR = 0.451, 95% CI = 0.265–0.766).

**Table 2:** The comparison of HCMV prevalence in CRC tissues

Category	Subcategory	No. of studies	Prevalence (%) (95% CI)	Adjusted OR^a^	*P* value
Overall	Total	11	27.5 (17.2−37.8)	−	
Region	Developing region	7	40.0 (34.8−45.1)	Ref	
	Developed region	4	10.0 (4.6−15.5)	0.257 (0.159−0.414)	< 0.01
Year of publication	After 2010	7	37.4 (27.3−47.4)	Ref	
	Before 2010	4	7.9(3.1−12.7)	0.488 (0.237−1.005)	< 0.01
type of samples	Fresh and frozen	8	31.2 (19.2−43.3)	Ref	
	Formalin-fixed	3	17.0 (−0.2−34.2)	0.451 (0.265−0.766)	< 0.01

95% CI: confidence interval, OR: odds ratio.

a Adjusted by region, year of publication and type of samples.

### HCMV infection and CRC risk

**Figure 3:**
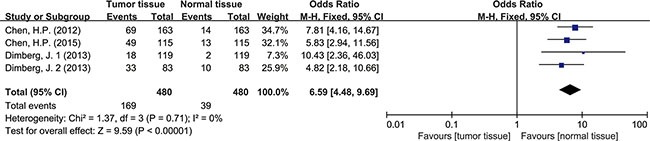
Forest plot of ORs for the association between HCMV infection and colorectal cancer risk

**Figure 4:**
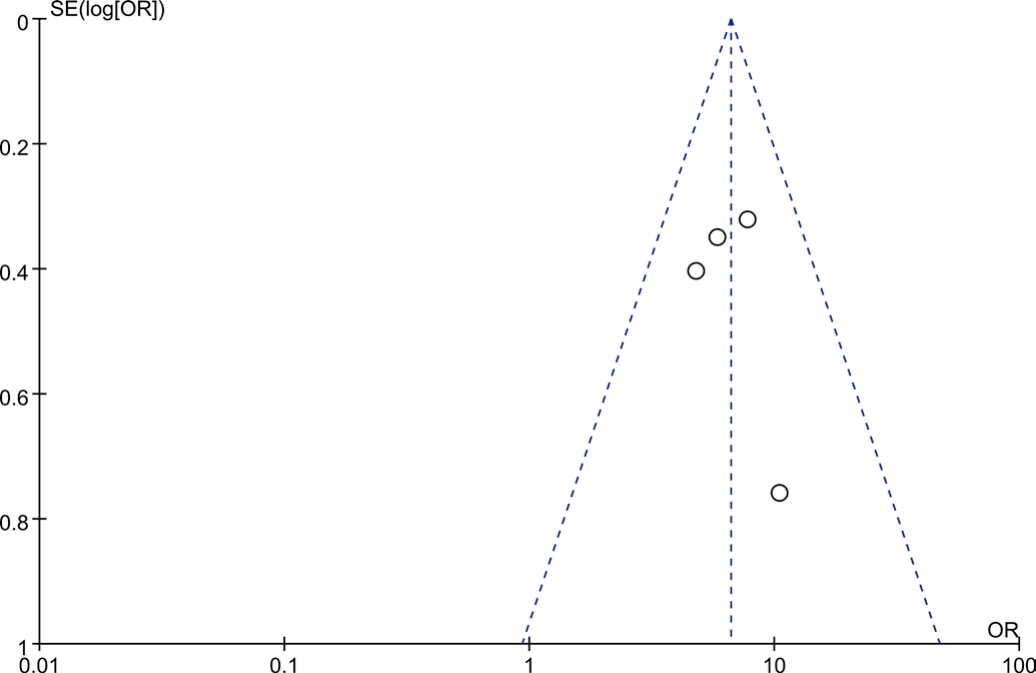
The funnel plots of the included studies OR, odds ratio; SE, standard error

Four articles studying tumor tissues with adjacent normal tissues from the same patients were selected for the meta-analysis. All of the 4 reports provided sufficient data to estimate the OR and 95%CI. The forest plot showed the effect size for each study and overall value (Figure 3). There was no significant heterogeneity across these studies (*I^2^* = 0%, *P* = 0.71), so a fixed-effects model was used to estimate the pooled OR. The result revealed that the tumor tissues had a significantly higher rate of HCMV infection (OR = 6.59, 95% CI = 4.48–9.69). No evident asymmetry of plots was seen in the funnel plots suggesting no evidence of publication bias (Figure 4).

### HCMV infection in tissues with different features

**Figure 5:**
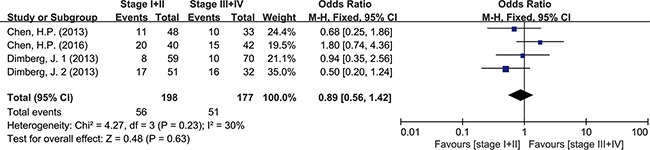
Difference in the tumor staging (stage I+II and stage III+IV)

**Figure 6:**
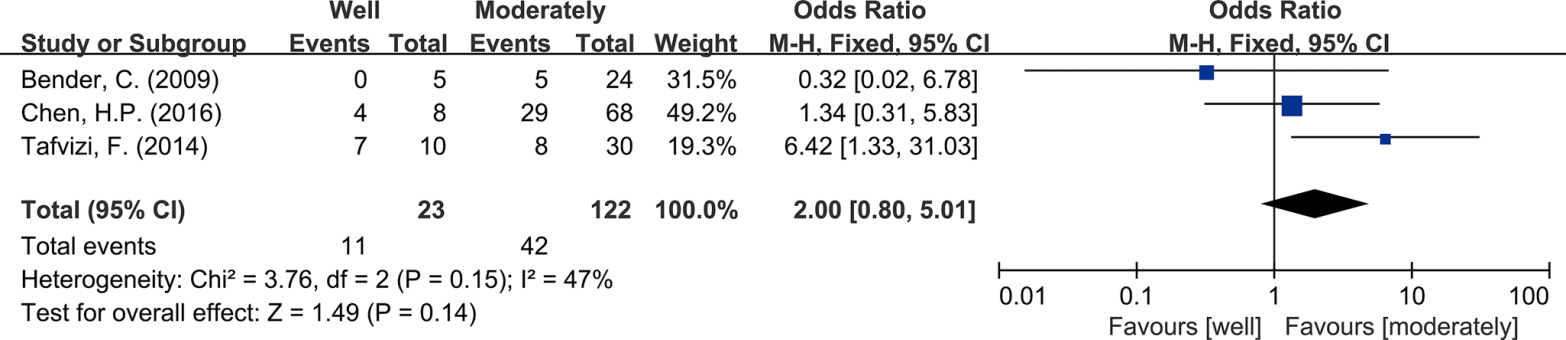
Difference in the grade of cell differentiation (well and moderately)

**Figure 7:**
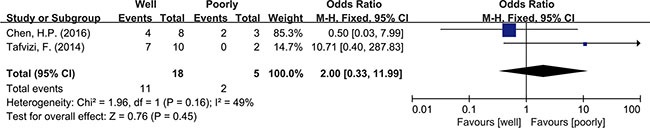
Difference in the grade of cell differentiation (well and poorly)

**Figure 8:**
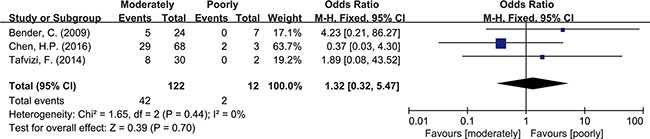
Difference in the grade of cell differentiation (moderately and poorly)

**Figure 9:**
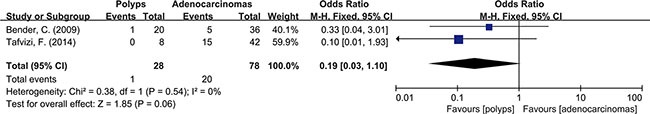
Difference in the tissues of polyp and adenocarcinoma

Meta-analysis was used to investigate more detailed data. Colorectal cancer was classified into 4 stages according to the American Joint Committee on Cancer (AJCC) classification system. There was no clear evidence indicating the difference of HCMV infection between different stages of cancer, such as subgroups of stage I+II and stage III+IV (OR = 0.89, 95% CI = 0.56–1.42) (Figure 5). In addition, HCMV infection was not related to tumor cells with different degree of differentiation. We compared the subgroups of well differentiated cells with moderately differentiated cells, also well with poorly and moderately with poorly. None of them revealed a significant difference in pooled data (OR = 2.00, 95% CI = 0.80–5.01; OR = 2.00, 95%CI = 0.33–11.99; OR = 1.32, 95% CI = 0.32–5.47, respectively) (Figures 6, 7, 8). There were two articles containing the data of HCMV detection in polyps and adenocarcinomas. The HCMV infection didn't statistically differ in tissues of polyp and adenocarcinoma (OR = 0.19, 95% CI = 0.03–1.10) (Figure 9).

## DISCUSSION

Over the past few decades, great attention had been paid to the relationship between HCMV infection and colorectal cancer. However the exact role of HCMV in patients with CRC still remained unclear. Among the majority of researches, the rate of HCMV detection in tumor samples represented the rate of HCMV-positive cancer, thus the influence of sample types and detection techniques being unneglectable. One single study with insufficient cases inevitably leaded to bias, which made a meta-analysis necessary.

Viral DNA and gene products including encoded RNAs and proteins were two general targets for viral detection. *In situ* hybridization (ISH) and immunohistochemistry (IHC) were available for the detection of gene products. However, as a latent virus, HCMV might not consistently express products during the whole period of infection, which would cause false-negative results and affect the accuracy of analysis [26, 27]. The PCR method was an important technique for DNA detection. PCR showed higher positive rate than ICH when a few studies combined both techniques of them. [19, 24] This controversy might result from the different detection techniques. Considering the bias of different methods, we chose studies which used PCR for HCMV DNA detection.

We found that there was a certain prevalence of HCMV DNA (28.7%) in CRC tissues, compared to a high rate of HCMV detection (over 80%) in patients’ plasma [16, 21]. Some reports attributed low or negative detection to the type of sample. Tissues processed by formalin would fail to do a detection of viral nucleic acids because of its effect on fragmentation of DNA. On the contrary, fresh and frozen sample was suitable for DNA preservation and detection [28, 29]. Here the data supported this opinion. DNA detection in fresh and frozen tissue showed more positive results than in formalin-fixed tissue (*P* < 0.01).

It had been reported that racial and geographic distribution of HCMV infection was existing [30, 31]. Here western and non-western countries shared a distribution of HCMV infection in CRC tissues (aOR = 0.257, 95% CI = 0.159–0.414), which revealed that there was less association between HCMV infection and CRC patients in developed than developing countries. This heterogeneity might be explained by the rate of HCMV infection and use of testing techniques and diagnostic criteria for HCMV infection in different regions. Epidemiological data suggested that sporadic CRC was more common in developed countries. Nevertheless the incidence in developing countries was increasing and it had been reported that the incidence of early onset CRC in developing was much higher [32, 33]. Whether HCMV played a role in these phenomena needed explaining by more data and further research. The prevalence of HCMV in colorectal cancer was significantly lower in the publication period before 2010 than after 2010 (aOR = 0.488, 95% CI = 0.237–1.005). The technical improvements and a larger sample size (700 cases versus 120 cases) might be a reason for the difference and made the results reported after 2010 more convincing. However negative results tended not to be published especially in the condition that negative results had already been published. Although the funnel plots indicated no publication bias, this could be an explanation, too.

Detection of HCMV DNA in CRC tissues by itself didn't provide adequate evidence to prove the causal role of HCMV in CRC. So data from 4 studies with total 480 tumor tissues and 480 matched normal tissues were pooled to give a more reliable evidence. Significant difference was seen in HCMV DNA prevalence between cancer and non-cancer tissue (OR = 6.59, 95% CI = 4.48–9.69). This observation supported the association of HCMV with colorectal tumor formation. The result refuted the conclusion that HCMV was not related to CRC risk, as some studies had drawn [19, 25]. For further analysis, we also pooled the data of different pathological features of tumor. The results indicated that HCMV seemed not to affect tumor staging and grade of differentiation since the detection of HCMV DNA showed no difference between stage I+II and stage III+IV, also no difference among high, moderate and low differentiations of tumor cell. As known to all, intestinal polyp, a precancerous lesion, could increase the risk of progression to colorectal cancer [34]. Our results showed no difference of HCMV infection in these two kinds of tissues. The results could be explained by one suggestion that HCMV infection might have influence on the every stage of tumor formation, not only on a single part. The detection of HCMV DNA might be a signal for malignant change of colorectal tissues but might not be an independent factor to predict the level of malignancy. However it had been reported that the distribution of HCMV proteins showed dramatic difference in tumor tissues (CRC or glioma) depending on the tumor histology [35, 36]. Viral non-coding RNAs regulatory proteins determined HCMV-induced oncomodulation thus affecting differentiation of tumor cells [37]. A further study about gene products of HCMV needed carrying out to provide a more specific explanation on tumor progression. Chen, et al. also reported that despite of TNM stage, HCMV in tumors were related to shorter disease-free survival in aged individuals with CRC [17]. It reminded us of other mechanisms that the virus had to affect the survival rate of patients.

It was important to realize the potential relationship between HCMV and CRC. This relationship could not only provide a deeper insight into the carcinogenic mechanism of CRC, but also offer a novel therapeutic option [38]. This was the first meta-analysis to investigate the association between HCMV infection and colorectal cancer. Moreover the parameters of geographical region, year of publication, type of samples, tumor staging, and histologic grade were evaluated in our study. These parameters could affect the detection of HCMV in CRC tissues and provide a basic knowledge for further analysis between HCMV or other viruses and CRC.

There were limitations in the present study. Our study focused only on researches related to DNA detection and didn't analyze the data concerning viral proteins detection. 7 articles were absent of control groups. In addition, none of included studies were random trials.

In conclusion, the present study indicated that a HCMV infection was statistically associated with an increased CRC risk and it played a role in the initiation and progression of CRC. Much further researches with larger scale of samples and more abundant date should be conducted to further confirm these results.

## MATERIALS AND METHODS

### Searching method and selection criteria

Literatures published up to January 2016 were searched in the electronic database of PubMed, Medline and EMBASE using the keywords “cytomegalovirus”, or “human herpesvirus-5” and “colorectal cancer” or “colorectal carcinoma”. The retrieved articles and their additional relevant references were reviewed meeting the following criteria: (1) Studies had to use polymerase chain reaction (PCR) technique to detect the presence of HCMV DNA in tumor tissues. (2) The articles should be with full text and published in English. *In vitro* studies based on cell lines or cells and review articles without original data were excluded.

### Data extraction

Data was extracted from the selected and qualified articles including the first author's name, the year of publication, geographic regions. The pathological characteristics of CRC specimen were also collected.

### Statistical analysis

The two main parameters analyzed for these studies were prevalence rate and odds ratio (OR). The statistical software OpenMeta-analyst (Center for Evidence-based Medicine, Brown University, Providence, R.I., USA) was used to analyze the proportions of samples positive for HCMV. The data from patient and control groups were pooled using Review Manager 5.3 (Cochrane Collaboration, Nordic Cochrane Centre, Copenhagen, Denmark). The meta-analysis was performed based on Mantel-Haenszel and DerSimonian-Laird methods according to heterogeneity which was tested by the *Q* statistic (significance level at *P* < 0.1) and the *I^2^* statistic (significance level at *I^2^* > 50%). If the *Q* or *I^2^* value was statistically significant, a random-effects model was adopted, otherwise a fixed-effects model was selected. Binary logistic regression was performed to compare and adjust the prevalence of HCMV by following factors: regions, the year of publication, detection techniques and specimen types with the adjusted OR (aOR) as an effect size. *P* < 0.05 were considered statistically significant.
